# Epidemiology of Back Pain in Children and Youth Aged 10–19 from the Area of the Southeast of Poland

**DOI:** 10.1155/2013/506823

**Published:** 2013-07-31

**Authors:** Agnieszka Kędra, Dariusz Czaprowski

**Affiliations:** ^1^Department of Posture Correction and Compensation, Faculty of Physical Education and Sport, Biala Podlaska 21-500, Jozef Pilsudski University of Physical Education, Warsaw 00-968, Poland; ^2^Department of Physiotherapy, Jozef Rusiecki University College, Olsztyn 10-243, Poland

## Abstract

*Objective*. The aim of this work was to define the prevalence of back pain in children and youth aged 10–19 from the southeast of Poland. *Material and Methods*. The cross-sectional study included 1089 students (547 girls and 542 boys) aged 10–19. The prevalence of back pain, its intensity, location, and situations in which it occurred were assessed with a questionnaire. *Results*. Among 1089 respondents, 830 (76.2%) admitted that they had experienced back pain at various frequencies within the year preceding the study. Back pain was located mainly in the lumbar segment (74.8%). Mild pains were dominant, which was declared by 44.7% of the respondents. Girls experienced back pain significantly more frequently than boys (52.2% versus 47.8%, *P* < 0.05). *Conclusions*. The research revealed that back pain is a common phenomenon. The prevalence of back pain in children and youth living in southeast Poland is similar to the frequency of occurrence of such complaints occurring in peers in other countries. It seems significant to monitor the remaining regions of Poland in order to define the scale of the problem and to look for the risk factors of back pain in children and youth to undertake efficient prophylactic actions.

## 1. Introduction

Musculoskeletal pains constitute a significant problem of the contemporary society [1]. One example of them is back pain [2]. Epidemiological data indicate that back pain is a significant problem not only in the group of adults but also among children and youth [3–8].

Potential risk factors influencing back pains include smoking tobacco [9, 10], diet [10], work [9, 11], sedentary lifestyle [12, 13], the level of physical fitness [10, 14], muscle elasticity and joints mobility range [15, 16], muscle strength [16, 17], school backpack weight [18–20], school furniture [21, 22], and psychosocial factors [14]. 

Back pain is not only a medical problem but also a socioeconomic problem. It may lead to limited functioning in everyday life, inability to work, and a bigger number of sick leaves [23, 24]. 

In the Polish scientific literature, there exist few epidemiological studies concerning back pain among children and youth in Poland. One of them which is worth mentioning is the work by Drozd et al. who conducted research in the group of 1475 students aged 13–20 from Poznan. The study revealed that back pain was experienced by 67% of all the subjects and recurring back pain was experienced by 49% of the general sample [25]. Romicka et al. examined 3386 children and youth aged 6–17 from Warsaw. Ten percent of the respondents reported back pain [26]. 

Due to the shortage of such epidemiological studies, the aim of the work was to define the frequency of occurrence and prevalence of back pain occurring in children and youth aged 10–19 from the area of the southeast of Poland. The frequency of occurrence of back pain (ranging from a single case to constant pain), its intensity, location, and situations in which it occurred were assessed.

## 2. Material and Method

The cross-sectional study included a group of 1224 students (547 girls and 542 boys) aged 10–19 attending 30 randomly selected state schools in 5 towns from the area of the south-east of Poland. The research sample was selected by means of two-stage cluster sampling [27]. At the first stage, schools representing three levels of education (primary, lower secondary, and upper secondary) were randomly selected in particular towns. At the second stage, years at schools were selected (stage cluster sampling) [27]. Eventually of the sample included pupils from years of 4–6 of primary school (children aged 10–13), years of 1–3 of lower-secondary school (14–16 years of age), and years of 1–3 of upper-secondary school (17–19 years of age). The number of schools was selected with the probability proportional to the number of children studying on three levels of education in particular towns. Data were gained from regional statistical offices, from reports prepared by the central statistical office, and from respective municipality Offices.

### 2.1. Diagnostic Tool

A questionnaire was used as a research tool. It was completed by all the students during school classes with one of the authors present. The first page of the questionnaire explained the aim of the research and included instructions for completing it. The questionnaire included multiple choice single-answer questions (6 questions) and multiple choice multiple-answer questions (29 questions). The multiple-answer questions additionally made it possible to add a comment after marking the option “other, what?”. The main part of the questionnaire concerned the following questions.

- Feeling or not back pain within the last year (12 months): people who responded negatively to this question did not complete the remaining part of the questionnaire.
- The location and frequency of the pain: in order to define the location of the pain reported by the subject, the questionnaire included drawings showing the location of cervical, thoracic, and lumbar segment of the spine.
- Ways of dealing with back pain (painkillers, contact with healthcare, and radiological examinations).
- Types of situations in which the first pain symptoms appeared.
- Situations in which back pain limited or prevented everyday physical activity.

In this work, only responses to these questions which were connected with the problems and objectives of the study were taken into consideration. 

### 2.2. Verification of the Tool

The questionnaire was tested in pilot studies in the group of 60 students representing all three stages of education (20 people at every stage of the three stages). In order to assess the reliability of the questionnaire, it was carried out in this group twice with a month break. The value of Kappa in all variables was equal or higher than 0.81 (0.81; 0.87; 0.88; 0.92; 0.93; 0.95; 0.95). No significant differences were revealed between the results obtained in the two tests (*P* < 0.05). 

### 2.3. Statistical Methods

Data were analysed by means of descriptive statistics and percent calculations. In order to test for differences between groups, a nonparametric test based on Chi-square function was used. Analysis of data was made with statistical and calculation programmes (SPSS 9.0, WINDOWS). Alpha *P* < 0.05 value was established as the level of the significance of differences.

The questionnaire was anonymous and voluntary, and a written consent was received from parents/legal guardians of children and from the Ethical Commission of Scientific Research of Jozef Pilsudski University of Physical Education in Warsaw, Poland. 

## 3. Results

Statistical analysis included 1089 questionnaires which were completed fully and correctly.  Table 1 presents the structure of age and gender of the subjects who completed the questionnaire correctly. 

### 3.1. The Frequency of Occurrence of Back Pain

Among 1089 respondents, 830 (76.2%) admitted that they had experienced back pain at various frequencies within the year preceding the study. This group included 52.2% of girls and 47.8% of boys. Having analysed the frequency of occurrence of back pain, it might be concluded that the biggest group was constituted by respondents who experienced pain rarely, that is, once or twice a year. The smallest group included students who experienced frequent or constant back pain. The number of people experiencing back pain very rarely (1-2 times per year), a few times per year (3–6), and very often or constantly did not increase with age. The analysis did not reveal a statistically significant correlation between age and the frequency of occurrence of back pain (very rarely, a few times per year, and frequently or constantly) (Table 2).

### 3.2. The Location of Back Pain

The question concerning the location of back pain was of a multiple-choice type. Back pain was mainly located in the lumbar segment. Such a location was reported by over 74.8% of girls and boys. The least common location of back pain was the thoracic segment of the spine, 11.1% of the respondents' answers (Table 2).

### 3.3. The Intensity of Back Pain

Having analysed the intensity of back pain, it was concluded that mild pain dominated, since it was reported by 44.7% of all the respondents. Nearly every fourth subject (22.5%) reported having experienced strong pain. It occurred in the group of 17–19 years old over twice more often than that in the group of 10–13 years old. The analysis revealed a statistically significant correlation between the intensity of back pain and the age of the subjects (*P* < 0.05) (Table 2).

### 3.4. Situations in which Pains Occurred and Ways of Dealing with Back Pain

The analysis of situations in which back pain occurred revealed that they included physical work (60.2%) and long-lasting sedentary position (35.4%) (Table 2).

Over 78.0% of the respondents do not take any actions to mitigate back pain. A significant number of students (12.3%) in all age groups mitigates pain with over-the-counter medicines. Only 40 respondents (4.8%) who reported experiencing back pain declared that they used various forms of physiotherapy as treatment (Table 3).

### 3.5. The Relation between Back Pain, Age, and Gender

The analysis revealed a statistically significant correlation between the subjects' age and the occurrence of back pain (*P* = 0.005). Back pain most frequently occurred in the oldest group of the examined children and youth (Table 4).

Another variable analysed was the relation between the occurrence of back pain and gender. It was revealed that girls experienced back pain significantly more frequently than boys (*P* = 0.02). The analysis revealed that the percentage of individuals experiencing back pain increases with age both in the group of girls (*P* < 0.05) and in the group of boys (*P* < 0.005). 

## 4. Discussion

The research revealed that the problem of the occurrence of back pain includes a big group (74.8%) of children and youth from the area of the south-east of Poland. The percentage of individuals (both boys and girls) experiencing back pain increases with age. Gender was a variable influencing the occurrence of back pain significantly. Girls experience back pain significantly more frequently. It seems interesting that despite the fact that in all age groups mild pain occurs most often, the number of people experiencing strong pain increases with age. Strong pain occurs twice more often in the group of 17–19 years old than in the group of 10–13 years old. Similar studies analysing back pain in children and youth were conducted in other countries. Research by Wedderkopp et al. carried out in Denmark revealed that back pain occurring in the period of a month before the date of the research was experienced by 39% of children and youth aged 8–10 and 14–16 [3]. In the American research by Sheir-Neiss et al. conducted among children aged 12–18, it was revealed that back pain occurred in 74.4% of the respondents [4]. In the research by Ayanniyi et al., it was revealed that back pain was reported by nearly 60% of children from Nigeria [28]. Differences in percentage values in the aforementioned studies concerning back pain may result from the fact that they examined different recall periods. Some studies took into consideration a one-month or two-month recall period while other authors analysed one-year (12 months) recall period. The study presented in this work referred to a one-year recall period, which may lead to the fact that the percentage of subjects who experienced back pain is higher. 

While comparing students from the three age groups, it was noted that the frequency of occurrence of back pain was increasing with the subjects' age. This tendency complies with earlier works which analysed the frequency of occurrence of back pain among children and youth in various countries [2, 23].

Back pain was mainly located in the lumbar segment, which was reported by over 74% of the respondents. The next most common location was the cervical segment (16.6%) and thoracic segment (11.1%) of the spine. No differences concerning the location of the pain between particular age groups were noted. A similar location of back pain as in the examined group of children and youth was revealed in the research by Wagenhauser, who claimed that back pain was usually located in the lumbar segment (53.5%) and then in the cervical segment (23.4%). The least common pain was that located in the thoracic segment (12%) [29]. In turn, the research carried out by Diepenmaat et al. indicated that back pain was usually located in the cervical segment and then in the lumbar segment [30]. In the research by Wedderkopp et al., it was concluded that back pain in children (8–10 years of age) occurred more often in the thoracic segment, while in the puberty period (14–16 years of age) the frequency of occurrence of back pain in the thoracic and lumbar segment was similar [3]. The obtained results also opposed the research by Vikat et al., which revealed that back pain is located in the cervical segment more often than in the lumbar segment [2]. It is hard to conclude firmly what the reasons for such big discrepancies concerning the location of the experienced back pain are. Nevertheless, according to Wedderkopp et al., in research on children and youth, back pain located in the cervical, thoracic, and lumbar spine should be analysed separately for clinical and scientific reasons [3].

The research revealed that physical work and long-lasting sedentary position were the main situations in which back pain occurred. 

It is interesting that over 78% of the respondents did not take up any action eliminating or mitigating back pain. It is also disturbing that a big group of respondents tried to mitigate the existing pain with over-the-counter painkillers. These observations are also confirmed by Burton et al., who concluded that only 15% of the subjects asked a physiotherapist or doctor for help [31].

### 4.1. Practical Applications

The research conducted in the group of children and youth from the area of the south-east of Poland revealed that back pain is a common phenomenon. It is significant for general practitioners, clinicians, and parents to be aware of the fact that back pain is quite common already in this age group. In the majority of cases it is mild or moderate pain. The percentage of children and youth with strong pain increases with age. 

It is also significant to conduct observations aiming at the identification of potential risk factors of back pain. Defining them may contribute to a more precise and accurate selection of prophylactic actions. Higher efficiency of prophylactic actions may, in turn, contribute to gaining not only medical but also psychosocial and economic profits. 

### 4.2. Study Limitations

Unfortunately, due to the study design we were not able to obtain reliable data on the individual physical activity and were therefore unable to correlate back pain with physical activity in children and youth under research.

## 5. Conclusions

The research conducted in the group of children and youth from the area of south-east Poland revealed that back pain is a common phenomenon. The pain is usually mild and located mainly in the lumbar segment of the spine. The percentage of individuals experiencing strong pain and the frequency of occurrence of back pain in general increase with age. Girls experience back pain significantly more frequently than boys.

## Figures and Tables

**Table 1:** Description of the study sample (*n* = 1089).

	Number	%
Distribution of the respondents according to age		
10–13 years	243	22.3
14–16 years	197	18.1
17–19 years	649	59.6
Gender distribution		
Girls	547	50.2
Boys	542	49.8

**Table 2:** The frequency of occurrence, location, intensity, and situations in which back pain occurs in the group of 10–19-year-old students according to age.

	Students with back pain (*n* = 830)		10–13 years (*n* = 156)		14–16 years (*n* = 143)		17–19 years (*n* = 531)	
	*n*	%	*n*	%	*n*	%	*n*	%
Frequency of occurrence of back pain (*P* > 0.05)								
Very rare back pain (1-2 times per year)	493	59.4	96	61.5	94	65.7	303	57.1
Back pain a few times per year (3–6 times per year)	231	27.8	41	26.3	29	20.3	161	30.3
Frequent or constant back pain (more than 1-2 times per month)	106	12.8	19	12.2	20	14.0	67	12.6
Girls with back pain	433	52.2	82	52.6	79	55.2	272	51.2
Boys with back pain	397	47.8	74	47.4	64	44.8	259	48.8
The location of back pain*								
Cervical segment	138	16.6	34	21.8	23	16.1	81	15.2
Thoracic segment	92	11.1	12	7.7	25	17.5	55	10.3
Lumbar segment	621	74.8	115	73.7	101	70.6	405	76.3
The intensity of back pain (*P* > 0.05)								
Mild	371	44.7	103	66.0	57	39.8	199	37.5
Moderate	272	32.8	32	20.5	47	32.9	159	29.9
Strong	187	22.5	21	13.5	39	27.3	173	32.6
Situations in which back pain occurs								
Physical work	500	60.2	101	64.7	89	62.2	310	58.4
Long-lasting sedentary position (over 6 h daily)	294	35.5	49	31.4	44	30.8	201	37.9
Stress	22	2.6	2	1.3	6	4.2	14	2.6
Other	14	1.7	4	2.6	4	2.8	6	1.1

*n*: number of people; %: percent value; *does not add to 100%, since the respondents were allowed to indicate more than one answer.

**Table 3:** Dealing with back pain.

	Students with back pain (*n* = 830)		10–13 years (*n* = 156)		14–16 years (*n* = 143)		17–19 years (*n* = 531)	
	*n *	%	*n *	%	*n *	%	*n *	%
Taking painkillers (pharmacological treatment)								
Prescribed by a doctor	37	4.5	10	6.4	10	7.0	17	3.2
Over-the-counter	102	12.3	26	16.7	15	10.5	61	11.5
Physiotherapy (manual therapy, electrotherapy, exercises)	40	4.8	8	5.1	4	2.8	28	5.3
No treatment	651	78.4	112	71.8	114	79.7	425	80.0

*n*: number of subjects; %: percent value.

**Table 4:** Correlation between back pain and age or gender.

	*n* = 830	%	*P* value
Correlation between age and back pain			
10–13 years (*n* = 243)	156	64.2	0.005
14–16 years (*n* = 197)	143	72.6	
17–19 years (*n* = 649)	531	81.8	

Correlation between gender and back pain			
Girls	433	52.2	0.022
Boys	397	47.8	

Correlation between girls' age and back pain			
10–13 years (*n* = 243)	82	72.6	0.05
14–16 years (*n* = 197)	79	73.8	
17–19 years (*n* = 649)	272	83.2	

Correlation between boys' age and back pain			
10–13 years (*n* = 243)	74	56.9	0.005
14–16 years (*n* = 197)	64	71.1	
17–19 years (*n* = 649)	259	83.8	

*n*: number of subjects; %: percent value.
